# Management of severe acute malnutrition by cow milk in resource constraints settings: experience of the Nutritional Centre of the University Clinics of Graben

**DOI:** 10.1186/s12887-018-1115-x

**Published:** 2018-04-20

**Authors:** Mupenzi Mumbere, F. Katsuva Mbahweka, B. P. Furaha Nzanzu

**Affiliations:** 1 Department of Paediatrics, University Clinics of Graben, Catholic University of Graben, Butembo, Democratic Republic of Congo; 2 Matanda Hospital, Butembo, Democratic Republic of Congo

**Keywords:** Severe acute malnutrition, Cow milk, Soybean, Maize, Vegetal oil, Sugar, Therapeutic milk

## Abstract

**Background:**

Severe acute malnutrition is defined as a weight for height z-score < − 3 standard deviation. Since 2000, joint efforts of the World Health Organization and United Nations Children’s Fund allowed to standardize the management of acute malnutrition by improving outcome and preventing complications with the introduction of therapeutic milk and ready-to-use therapeutic foods. However, in the Democratic Republic of Congo, many health facilities face therapeutic milk shortage while managing severe acute malnutrition. At the University Clinics of Graben, cow milk with porridge made of maize, soybean, vegetal oil and sugar is used during stockouts periods. This study was carried out to analyse the efficiency and safety of this treatment compared to the conventional one in SAM patients.

**Methods:**

This study is based on the experience of the University Clinics of Graben in eastern Democratic Republic of Congo whose nutritional centre is often confronted with stockouts in nutritional supplements. During a three months shortage in 2015, patients received cow milk alternating with preparations made from sugar-maize-soybean- vegetal oil. The study compared the evolution of these children with those who had previously been treated with the WHO conventional preparations by analysing weight changes, oedema resolution, gastrointestinal tolerability and clinical outcome over 21 days. Data were analysed with SPSS 20. We used the ANOVA, Chi-square test, odd ratio and *p*-value to compare the differences.

**Results:**

Seventy-nine patients had received cow milk while fifty-seven were submitted to classical therapeutic milk. There was no significant difference between the two groups regardless the type of malnutrition in terms of weight changes, oedema resolution, gastrointestinal tolerability and clinical outcome over 21 days.

**Conclusion:**

Cow milk alternately with sugar-maize-soybean- vegetal oil preparations is an acceptable alternative in case of stockouts in conventional therapeutic milk in these settings.

## Background

Severe acute malnutrition (SAM) is defined as a z-score weight-for-height (W/H) below − 3 standard deviation. Two major clinical forms are generally recognized: kwashiorkor which is an oedematous malnutrition and marasmus which is a significant emaciation with a mid-upper arm circumference (MUAC) < 115 mm. In between, mixed malnutrition is described [1]. Each year, SAM affects at least 19 million under-5 children globally. In these children, the risk of mortality is ten folds higher than among those whose z-score weight-for-height is> − 1 [2]. Malnutrition is responsible of the deaths of nearly half of under-five children [3].

Since 2000, joint efforts of the World Health Organization (WHO) and the United Nations Children’s Fund (UNICEF) allowed the standardization of the management of acute malnutrition which improved outcome and prevented complications. Ready-to-use therapeutic foods and therapeutic milk enriched in macro and micronutrients as well as the implementation of community-based malnutrition management have brought enormous progress [4]. Between 2001 and 2010, the Democratic Republic of Congo (DRC) experienced a significant improvement in diet and malnutrition rate fell down from a national prevalence of 16 to 11% for all ages [5].

In DRC, nutritional management of moderate acute or uncomplicated severe malnutrition is based on the ready to use therapeutic foods (RUTF) which consist of dry rations containing a mixture of legumes and starchy foods fortified with micronutrients, oil, vitamin A and sugar. They assure between 1000 and 1200 Kcal / day / person. In complicated severe acute malnutrition, the management is based on therapeutic milk (TM) F75 and F100, which is the core of the intervention. The last two provide an energy contribution between 100 and 150 Kcal / kg / day [5]. F-75, a low-protein milk-based formula diet, is given as the therapeutic food in the stabilization phase, while F-100, a milk formula with higher protein and energy content, is recommended as the therapeutic food in the rehabilitation phase [6].

DRC is a huge 2,345,000 km^2^ country with a health system mainly based on foreign partners’ assistance. Regarding nutritional care, the National Nutrition Programme (PRONANUT) supported by UNICEF provides supplies to health facilities in therapeutic consumables. However, many frequent stockouts due to the weakness of the national distribution system are hampering the implementation of WHO recommendations [7–9].

The Nutritional Therapeutic Intensive Unit (UNTI) Giorgio Cerruto is hosted by the University Hospital of Graben, located in the city of Butembo, North Kivu province in eastern DRC. Children with SAM are treated in accordance with the National Protocol for Integrated Management of Malnutrition (PCIMA), inspired from the WHO recommendations. However, this nutritional therapeutic unit is often confronted to the stockouts in therapeutic supplies (RUTF, F75 and F100). So, health care providers are obliged to use cow’s milk (CM), sugar, vegetal oil, maize and soy porridge preparations (MASO) in SAM management during the periods of shortage. This preparation is constituted of fresh cow milk (80 g), sugar (65 g), Rina^R^ vegetable oil which is derived from palm oil (20 g) and cereals (35 g).

The objective of this study is to evaluate the efficacy and safety of the combination CM / MASO, vegetable oil and sugar in the management of severe acute malnutrition compared to conventional WHO intervention based on TM / RUTF.

## Methods

### Study site

The study was conducted in the Paediatric Service of the University Hospital of Graben in the UNTI Giorgio Cerruto, with a capacity of 30 beds. The Centre admits an average of 22 cases of severe malnutrition monthly.

### Study type and population

We conducted a retrospective comparative study on the records of patients who were followed up for SAM. Patients with complete records were included in the analysis. Those with incomplete records were excluded. 136 patients met the inclusion criteria and constitute the study sample. Of these, 57 patients had received therapeutic milk F75, F100 and RUTF (LT / RUTF group) for a three months period prior to the stockout (January–March 2015) and 79 were administered cow milk / vegetal oil-sugar-maize-soybean (CM/MASO group) for three months (April–June 2015) when the UNTI was out of stock in conventional therapeutic supplies.

### Data collection and study parameters

Data were collected from the individual patient records and entered in an electronic database in SPSS 20. For each patient, the following variables were considered: age, sex, nutritional diet, weight, size, presence of oedema, signs of intolerance, diagnosis, comorbidity and clinical outcome.

### Evaluation criteria

We compared weight changes, oedema resolution at Day (D) 1 (baseline), D3, D5, D7, D14 and D21, the tolerance of the respective regimens and the clinical outcome in both groups.

Weight was measured in kilograms; oedema resolution in number of crosses (3 crosses for generalized oedema; two crosses for bilateral oedema of the feet, ankles and legs, hands and forearms; one cross for the bilateral feet oedema [6] and tolerance by the occurrence of diarrhoea, vomiting or constipation in the week following the initiation of nutritional diet [10]. Clinical outcome was assessed as “improved”, “not improved” or “died”, based on attending physician’s judgement which was recorded on patients’ files.

### Data processing and analysis

Data were checked for consistency and completeness by matching the source records, the data collection form and the electronic database by a double independent verification process and were analysed using SPSS 20 software.

For continuous values, we used means and standard deviations. We used the analysis of variance (ANOVA) to compare the weight means changes between the groups with the F test of Fisher. Where needed and if applicable, we used the Chi-square test, the odds ratio or relative risk and *p*-values to compare the considered parameters in the two groups. For odds ratio and relative risk, we calculated the confidence intervals. The level of significance was 0.05.

### Ethical considerations

Our study protocol was approved by the Ethics Committee of North-Kivu. As we only searched data from the records of previously treated patients, risk was deemed minimal for them. All personal identification information was coded in the database. Source documents were accessible only by assigned and authorized staff as the research team routinely abides to the ethical duty of medical confidentiality.

## Results

### Demographic characteristics of the study population

We analysed the records of a total of 136 patients including 65 females (47.8%) and 71 males (52.2%). In these, 96 (70.6%) had kwashiorkor; 22 (16.2%) marasmus and 18 (13.2%) mixed severe acute malnutrition. The median age was 24 months for the CM / MASO group (minimum age 8 months; maximum age 204 months); 36 months for the TM / RUTF group (minimum age 3 months, maximum age 144 months). At baseline, median weight was 8.4 kg in the cohort CM / MASO (minimum 4.1 kg; Maximum 17 kg); 9.4 kg cohort in TM / RUTF (minimum 1.7 kg; maximum 18 kg) (Table 1).

**Table 1:** Demographic characteristics of the study population

Age groups in months	F	M	Total	Kwashiorkor			
1–12	8	11	19	Nutritional management	F	M	Total
13–24	16	31	47	CM/MASO	26	24	50
25–36	14	10	24	TM/RUTF	22	24	46
37–48	13	8	21	Total	48	48	96
49–60	4	6	10	Mixed malnutrition			
> 60	10	5	15	CM/MASO	5	9	14
Total	65	71	136	TM/RUTF	2	2	4
Weight groups in Kg				TotaL	7	11	18
1–5	8	11	19	Marasmus			
6–10	39	41	80	CM/MASO	8	7	15
11–15	15	15	30	TM/RUTF	2	5	7
> 15	3	4	7	TotaL	10	12	22
Total	65	71	136	TOTAL	65	71	136
Height groups in cm							
≤60	3	4	7				
61–80	37	38	75				
81–100	20	20	40				
> 100	5	9	14				
Total	65	71	136				

### Weight trend in both groups

**Fig. 1:**
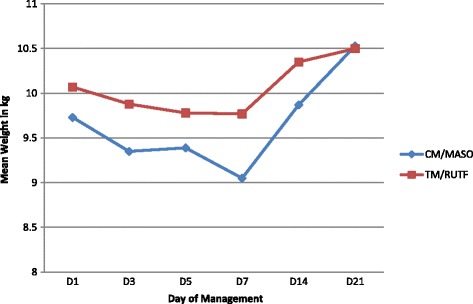
Kwashiorkor patients weight graph over 21 days

**Fig. 2:**
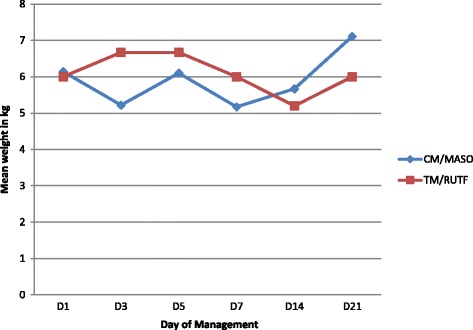
Marasmus patients weight graph over 21 days

**Fig. 3:**
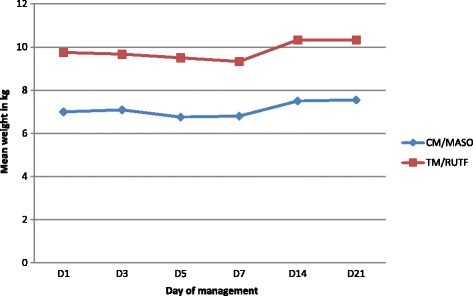
Mixed malnutrition patients weight graph over 21 days

This section gives the comparison of means of weight in each type of malnutrition and nutritional management. The evolution is given at D1, D3, D5, D7, D14 and D21. The results for patients with Kwashiorkor are showed in Table 2 with its graph in Fig. 1; the ones for patients with marasmus in the Table 3 and Fig. 2 and for the patients with mixed malnutrition in the Table 4 and Fig. 3.

**Table 2:** Daily mean weight evolution in kwashiorkor children over 21 days

Nutritional management		D1	D3	D5	D7	D14	D21
CM/MASO	N	51	40	44	44	46	34
	Mean weight in Kg	9.73	9.35	9.39	9.05	9.87	10.53
	SD	2.850	2.966	2.895	2.885	2.895	2.957
	Min weight in Kg	5	5	5	5	6	6
	Max weight in Kg	17	17	16	16	16	16
	Weight gain/loss in Kg		−0.38	−0.34	−0.68	+ 0.14	+ 0.8
TM/RUTF	N	45	43	41	43	40	34
	Mean weight in Kg	10.07	9.88	9.78	9.77	10.35	10.50
	SD	3.026	3.033	3.078	2.894	3.093	3.422
	Min weight in Kg	5	5	4	5	5	6
	Max weight in Kg	18	18	18	18	17	19
	Weight gain/loss in Kg		−0.19	−0.29	−0.3	+ 0.02	+ 0.43
Total	N	96	83	85	87	86	68
	Mean weight in Kgs	9.89	9.63	9.58	9.40	10.09	10.51
	SD	2.923	2.995	2.974	2.895	2.981	3.174
	Min weight in Kg	5	5	4	5	5	6
	Max weight in Kg	18	18	18	18	17	19
	Weight gain/loss in Kg		−0.26	−0.31	− 0.49	+ 0.20	+ 0.65
Test F ANOVA		0.323	0.655	0.370	1.358	0.553	0.001
df		95	82	84	86	85	67
*p*-value		0.571	0.421	0.545	0.247	0.459	0.970

**Table 3:** Daily mean weight evolution in marasmus children over 21 days

Nutritional management		D1	D3	D5	D7	D14	D21
CM/MASO	N	14	9	10	6	12	9
	Mean weight in Kg	6.14	5.22	6.10	5.17	5.67	7.11
	SD	2.538	1.202	3.315	0.753	0.985	3.551
	Min weight in Kg	4	4	4	4	5	5
	Max weight in Kg	14	8	15	6	8	16
	Weight gain/loss in kg		−0.92	−0.04	−0.97	−0.47	+ 0.97
TM/RUTF	N	8	6	6	7	5	6
	Mean weight in Kg	6.00	6.67	6.67	6.00	5.20	6.00
	SD	3.266	3.327	3.327	3.512	2.588	2.898
	Min weight in Kg	2	2	2	2	2	2
	Max weight in Kg	11	12	12	12	8	9
	Weight gain/loss in kg		+ 0.67	+ 0.67	0	−0.80	0
Total	N	22	15	16	13	17	15
	Mean weight in Kg	6.10	5.80	6.31	5.62	5.53	6.67
	SD	2.719	2.305	3.219	2.567	1.546	3.244
	Min weight in Kg	2	2	2	2	2	2
	Max weight in Kg	14	12	15	12	8	16
	Weight gain/loss in Kg		−0.30	+ 0.21	−0.48	−0.57	+ 0.57
Test F ANOVA		0.012	1.460	0,.09	0.321	0.308	0.404
df		20	14	15	12	16	14
*P*-value		0.913	0.248	0.746	0.746	0.587	0.536

**Table 4:** Daily mean weight in mixed malnutrition children over 21 days

Nutritional management		D1	D3	D5	D7	D14	D21
CM/MASO	N	13	11	12	10	12	11
	Mean weight in Kg	7.00	7.09	6.75	6.80	7.50	7.55
	SD	2.160	1.868	1.913	1.687	1.883	2.207
	Min weight in Kg	4	4	4	5	5	5
	Max weight in Kg	11	10	10	10	10	11
	Weight gain/loss in Kg		+ 0.09	−0.25	−0.20	+ 0.50	+ 0.55
TM/RUTF	N	5	3	4	3	3	3
	Mean weight in Kg	9.75	9.67	9.50	9.33	10.33	10.33
	SD	3.304	4.041	3.317	4.509	4.509	5.132
	Min weight in Kg	6	6	6	5	6	6
	Max in Kg	14	14	14	9	15	16
	Weight gain/loss in Kg		−0.08	−0.25	−0.42	+ 0.58	+ 0.58
Total	N	18	14	16	13	15	14
	Mean weight in Kg	7.65	7.64	7.44	7.38	8.07	8.14
	SD	2.644	2.530	2.529	2.599	2.658	3.035
	Min weight in Kg	4	4	4	5	5	5
	Max weight in Kg	14	14	14	14	15	16
	Weight gain/loss in kg		−0.01	−0.21	−0.27	+ 0.42	+ 0.49
Test F ANOVA		3.910	2.777	4.336	2.458	3.144	2.168
df		16	13	15	14	14	13
p		0.067	0.121	0.056	0.145	0.100	0.167

Table 2 shows that patients with Kwashiorkor in CM / MASO group gained an average of 0.8 Kg from D1 (9.73 Kg) to D21 (10.53 Kg). In the TM/ RUTF group, the mean weight rose from 10.07 Kg at D1 to 10.51 kg at D21 (mean weight gain 0.43 kg). After the Anova test, the observed differences were not statistically significant. The calculated F test were less than the tabular F test with *P*-value more than 0.05.

Table 3 shows that children with marasmus in the CM / MASO group gained a mean weight of 0.97 kg from D1 (6.14 kg) to D21 (7.11 kg). In the TM / RUTF group, the average weight did not change from D1 to D21.

Table 4 shows that the children with mixed malnutrition in the CM / MASO group had a mean of 7 kg at D1 and 7.55 kg at D21 which represented a mean weight gain of 0.55 kg. In the TM / RUTF group, the mean weight was 9,75Kg at D1, 10,33 kg at D21 which represented a weight gain of 0,58 kg. After the Anova test, these differences were not significant. The *P* values were all more than 0.05.

### Resolution of oedema

Kwashiorkor or mixed malnutrition are recognized for bilateral oedema which may be severe (+++), moderate (++) or mild (+).

**Fig. 4:**
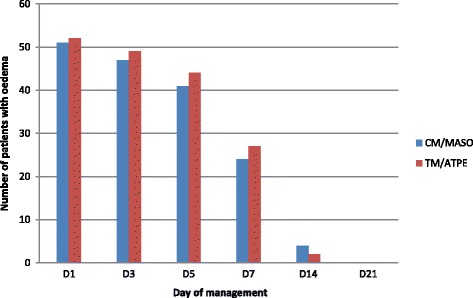
Number of oedematous patients over 21 days

In both groups, oedema resolved the same way (Table 5, Fig. 4). On day 7, half of initially oedematous patients had recovered. On D14, four patients still remained oedematous in the CM / MASO cohort. Their comorbidities were: gastroenteritis (two), pneumonia and intestinal parasitosis (one); urinary tract infection and intestinal parasitosis (one). In the TM / RUTF cohort, two patients were still oedematous. Their comorbidities were: intestinal parasitosis (one); tuberculosis, chronic enteritis and trophic ulcers on a psychomotor retardation background (one). On D21, no patient was oedematous. We conducted a Student t test to check the homogeneity of the two groups. The calculated T is 0.950 with 1.96 as T theoretical. No significant difference between the two treatment groups was found.

**Table 5:** Oedema resolution

Type of management	Number of oedematous patients											
	D1	%	D3	%	D5	%	D7	%	D14	%	D21	%
CM/MASO	51	64,6	47	59,5	41	51,9	24	30,4	4	5,1	0	0
TM/RUTF	52	91,2	49	86,0	44	77,2	27	47,4	2	3,5	0	0

### Digestive tolerance of nutritional diets

Tolerance of regimen was defined by the occurrence or not in the week following the initiation of the nutritional management of one or more of the following symptoms: diarrhoea, vomiting and constipation (Table 6).

**Table 6:** Digestive tolerance in both nutritional regimens

Symptom	Nutritional management	N	Case	%	X^2^	df	p	RR	<limit	>limit
Diarrhoea	CM/MASO	80	23	28,8	0,517	1	0,471	1,23	0,68	2,23
	TM/RUTF	56	13	23,2				0,81	0,44	1,45
Vomiting	CM/MASO	80	15	18,8	0,163	1	0,687	1,16	0,54	2,47
	TM/RUTF	56	9	16,1						
Constipation	CM/MASO	80	1	1,2	0,065	1	0,798	0,70	0,04	10,96
	TM/RUTF	56	1	1,8				1,43	0,09	23,46

Diarrhoea was the most presented symptom in the week following initiation of treatment (28.8% in the CM/MASO group and 23.2% in the TM/RUTF group), followed by vomiting (18.8% in CM/MASO group and 16.1% in the TM/RUTF group). Constipation was very rare in both groups. After a Chi-square analysis, we found no significant difference in the occurrence of digestive symptoms in the two groups (*p* > 0.05).

### Clinical outcome

We recorded two fatal cases (one in each group); two patients did not improve in the CM/MASO cohort. They presented the following comorbidities: gastroparesis (one), severe cerebral palsy (one). There was no significant difference in the two groups regarding clinical outcome.

## Discussion

This study attempted to understand whether the use of cow milk alternately with the preparations of maize-soybean-vegetal oil-sugar could be an alternative to conventional preparations recommended by WHO based on an observation from the Therapeutic Nutritional Centre Giorgio Cerruto experience at the University Clinics of Graben which was confronted to the shortage of nutritional consumables. Four parameters enabled us to achieve our goal: weight changes in each type of malnutrition, nutritional oedema resolution, gastrointestinal tolerability and clinical outcome [11].

### Weight change

Nutritional rehabilitation is based on weight control until a weight-for-height z-score > − 1 is reached or the MUAC is > 125 mm [1]. When we compared the averages of weight change in both cohorts regarding each type of malnutrition, mean weight gain was similar. Kwashiorkor patients first dropped down due to oedema resolution before increasing their weight from D7 while the patients with marasmus and mixed malnutrition had stable weight the first week before increasing the following weeks. We are not able to confirm that children recovered normal weight since data of weight-for-height z-scores and MUAC were missing for D21 and the period considered for analysis was too short as WHO recommends that children with severe acute malnutrition should only be discharged from treatment when their weight-for-height is ≥ − 2 Z-score and the MUAC is ≥125 cm and they had no oedema for at least 2 weeks [6]. Therefore, the follow up period for SAM management can go up to six weeks for full recovery. Nguefack and collaborators presented similar results in their study conducted at the University Hospital of Yaounde on the hospital management of severe acute malnutrition in children with local preparations made from whole milk (Nido®), cooking oil, sugar, cereals and multivitamins [12]. In Basra (Iraq), Sawsan I. Habeeb also found similar results in a retrospective study that evaluated the therapeutic effectiveness of F75 and F100 prepared locally (dried whole milk, cereals flour, sugar, vegetable oil and minerals) in comparison with industrially processed milk [13].

### Oedema resolution

Nutritional oedema is a common symptom in kwashiorkor and mixed malnutrition. It predicts for severity [5, 6, 14–16]. Their resolution is therefore a good surrogate for a positive response to nutritional management [17].

In both groups in our study, oedema resolved at a similar rate (Fig. 4). At the end of the first week of treatment, half of oedematous patients had resolved. At the end of the second week, six patients (four in the cohort CM / MASO and two in the TM / RUTF cohort) were still oedematous and at the end of the third week, no patient presented oedema. The six who still retained oedema at D14 had the following comorbidities: pneumonia, gastroenteritis, digestive parasitosis, urinary tract infection, cerebral palsy, sepsis and tuberculosis. Dominique Roberfroid and his collaborators in a systematic review of oedematous malnutrition management had also found that concomitant infections are among the risk factors of mortality and treatment response delay [17].

### Digestive tolerance of nutritional diets

While diarrhoea is a common symptom and a criterion of severity in SAM [18, 19], it is also a sign of digestive intolerance due to malabsorption alongside vomiting and constipation [11]. In both cohorts, tolerability was similar (*p* > 0.05) characterized by diarrhoea in a quarter of patients and vomiting in less than a fifth. Constipation was rare. Razafindrakoto and his colleagues in their study that compared goat with cow milk in the management of SAM also found that patients did not encounter digestive intolerance with cow milk [10].

### Clinical outcome

We recorded two deaths (1.5% total mortality rate, 1.3% in the CM / MASO group; 1.8% in the TM / RUTF group). The overall improvement rate was 97% (CM / MASO 96.3%; TM / RUTF 98.2%). Lower recovery rates (33.6%) were found in Tamale Teaching Hospital (Ghana) in a retrospective chart review study [20]. And a bigger mortality rate (3.7%) was recorded in Niger when evaluating a nutritional rehabilitation program, in patients with severe malnutrition treated according to the WHO standard procedures [21] while it ranged from 3.4 to 35% in a systematic review and meta-analysis of the management of severe acute malnutrition in low and middle income settings [22]. The reported high improvement and low mortality rates in our study may be due to the short study period.

### Limitations

Data on patients’ MUACs and weight for height z-scores were missing for D21. So we were unable to assess patients’ clinical outcome objectively. To address this limitation, we relied on the physician’s final judgement at patient’s discharge which was recorded on patients’ files. We were also unable to present a thorough discussion of the results because not many research works have been published on the comparison between WHO recommended therapeutic milks and locally processed therapeutic foods to treat SAM.

## Conclusion

Cow milk alternately with the preparations maize-soybean-vegetal oil-sugar is an acceptable alternative to conventional formulations of therapeutic milk (F75, F100 and RUTF). This locally accessible diet, adequately addresses the shortages in conventional supplies. Even though shortages are to be strongly discouraged; still, they are common in DRC, a subcontinent lacking an adequate health system.

## Abbreviations

ANOVA: Analysis of variance
CM: Cow milk
DRC: Democratic Republic of Congo
MASO: Maize-soya beans
MUAC: Mid-upper arm circumference
PCIMA: National Protocol for Integrated Management of Malnutrition
PRONANUT: National Nutrition Programme
RUTF: Ready to use therapeutic foods
SAM: Severe acute malnutrition
SPSS: Statistical package for the social sciences
TM: Therapeutic milk
UNICEF: United Nations Children’s Fund
UNTI: Nutritional Therapeutic Intensive Unit
WHO: World Health Organization
